# Selection against tandem splice sites affecting structured protein regions

**DOI:** 10.1186/1471-2148-8-89

**Published:** 2008-03-21

**Authors:** Michael Hiller, Karol Szafranski, Klaus Huse, Rolf Backofen, Matthias Platzer

**Affiliations:** 1 Bioinformatics Group, Albert-Ludwigs-University Freiburg, Georges-Koehler-Allee 106, 79110 Freiburg, Germany; 2 Genome Analysis, Leibniz Institute for Age Research – Fritz Lipmann Institute, Beutenbergstr. 11, 07745 Jena, Germany

## Abstract

**Background:**

Alternative selection of splice sites in tandem donors and acceptors is a major mode of alternative splicing. Here, we analyzed whether in-frame tandem sites leading to subtle mRNA insertions/deletions of 3, 6, or 9 nucleotides are under natural selection.

**Results:**

We found multiple lines of evidence that the human protein coding sequences are under selection against such in-frame tandem splice events, indicating that these events are often deleterious. The strength of selection is not homogeneous within the coding sequence as protein regions that fold into a fixed 3D structure (intrinsically ordered) are under stronger selection, especially against sites with a strong minor splice site. Investigating structures of functional protein domains, we found that tandem acceptors are preferentially located at the domain surface and outside structural elements such as helices and sheets. Using three-species comparisons, we estimate that more than half of all mutations that create NAGNAG acceptors in the coding region have been eliminated by selection.

**Conclusion:**

We estimate that ~2,400 introns are under selection against possessing a tandem site.

## Background

Many genes in animal and plant genomes express more than one transcript by alternative splicing. These transcripts can encode proteins with different, sometimes even antagonistic functions. For example, many members of the human caspase gene family express alternative splice variants that encode pro- and anti-apoptotic proteins [1]. In mammals, most alternative splice events skip complete exons or utilize either alternative donor or acceptor splice site pairs. Most of the latter (called tandem splice sites in the following) are in close proximity [2-6], thus leading to the insertion/deletion (indel) of only a few nucleotides. Alternative splice events at NAGNAG acceptors are the most frequent of these events. Previous studies suggest that the splicing mechanism of short-distance tandem sites involves stochastic selection of either site [7]. A subset of these events is under purifying selection, thus contributing to the repertoire of biologically relevant alternative splice events [8].

In humans, a substantial number of alternative splice events either shift the protein reading frame or directly introduce a premature termination codon by skipping of exons or alternative usage of tandem splice sites [3,9-11]. Most of these events render the transcript a target for nonsense-mediated mRNA decay (NMD) [11]. Conservation of such events implies functionality, maybe by regulating the protein level [8,12-14]. On the other hand, many of these events likely have no functional relevance or may be due to splicing errors [15]. Here, NMD is an important surveillance mechanism reducing the amount of transcripts that would be translated to truncated proteins [16].

Apart from NMD, cells can use the complex splicing regulatory mechanisms to silence deleterious splice events. For example, pseudo exons (silent intronic regions that resemble real exons) are enriched in binding sites for silencing splicing factors, which prevent their inclusion into the mature transcript [17]. Likewise, silencer motifs located between two alternative splice sites inhibit the use of one splice site [18]. Thus, auxiliary splicing enhancer and silencer signals enable a high splicing fidelity, despite the occurrence of numerous pseudo splice sites. Consequently, there seems to have been no need to get rid of all these pseudo splice sites in the course of evolution.

This situation is different for deleterious short-distance tandem splice sites that preserve the reading frame. Firstly, they do not elicit NMD and secondly, mechanisms to inhibit the use of the alternative splice site seem to be limited. The latter reason is due to spatial restrictions that do not allow the placement of splicing silencer motifs between two splice sites separated by as few as 3 nucleotides (nt).

Furthermore, core components of the spliceosome are likely to be the major factors that enable such alternative splice events, whereas other splice events often depend on additional enhancing and/or silencing splicing factors. This makes tandem splice events rather independent of tissue-specific fluctuations in splicing factor concentrations. Consistently, many tandem sites produce constant splice variant ratios [19-21], although variation in the ratio was observed in several cases [4,20,22-24]. Assuming that splicing regulatory mechanisms in general cannot completely inhibit alternative splicing at these sites, the ultimate option to get rid of a deleterious tandem splice event is to destroy the tandem site, for example by a mutation that destroys one GT/GC donor or one AG acceptor dinucleotide. Under this assumption, we would expect to find traces of natural selection, evident as an underrepresentation of tandem splice sites at places where they are deleterious.

To test this hypothesis, we analyzed frame-preserving tandem splice sites with a distance of 3, 6, and 9 nt (Δ3, Δ6, and Δ9 nt, respectively). We focused on short-distance sites because a stochastic splicing mechanism is likely to be the basis for such alternative splice events [7]. We present multiple lines of evidence that such tandem sites are underrepresented in protein-coding sequences (CDS), and in particular in regions that form ordered 3D protein structures. We estimate that ~2,400 introns are under selection against possessing a tandem site.

## Results

### Underrepresentation of tandem splice sites in coding regions

We used human RefSeq transcript exon-intron structures and a series of stringent filtering steps (see Methods) to create a data set of 15,511 protein coding genes. Each gene is represented by a single transcript. These genes contain 140,975 introns that reside within the CDS and 9,077 introns in the 5' or 3' untranslated region (UTR). In the following, we analyze Δ3, Δ6, and Δ9 tandem donor and acceptor sites, where the occurrence of alternative splicing could be inferred from mRNA/EST data.

**Figure 1:**
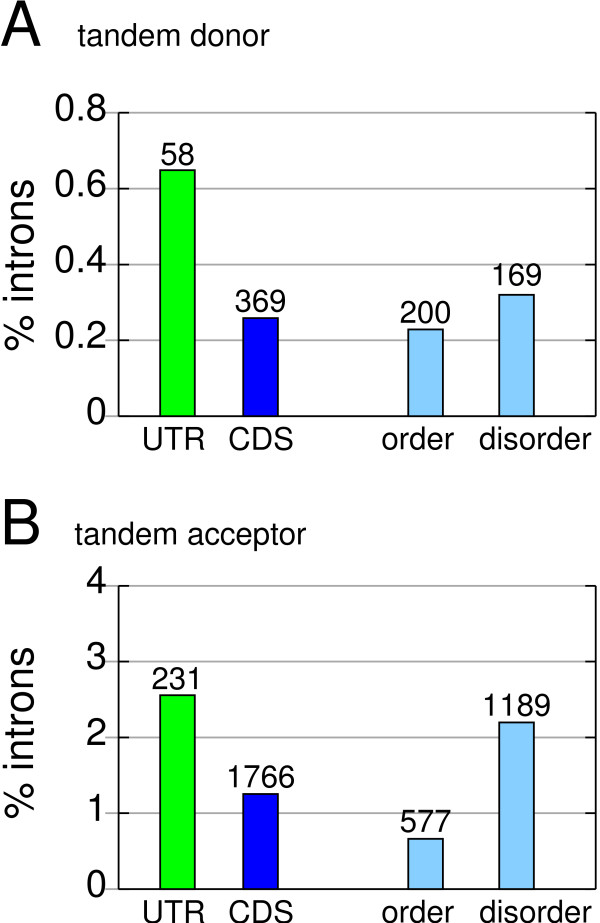
Frequency of tandem splice sites in the CDS and UTR. Each bar is the percentage of human introns having a tandem donor (A) or acceptor (B). Introns are divided into a location in the CDS (blue) and the UTR (green). CDS introns are further divided into a location in ordered and disordered protein regions (light blue). Absolute numbers are given above the bars.

We found that 0.26% of the CDS introns have a tandem donor (100, 142, 127 for Δ3, Δ6, Δ9, respectively) and 1.25% a tandem acceptor (1,396, 238, 132). In contrast, UTR introns, where such subtle events are expected to be neutral or only slightly deleterious, have a more than 2-fold higher fraction of tandem donors and acceptors (0.64% and 2.54%, respectively, Fisher's exact test: P < 0.0001, Figure 1). This suggests a general underrepresentation of tandem splice events in coding regions and is consistent with a report for NAGNAG sites [7].

### Inhomogeneous distribution of tandem sites in ordered and disordered protein regions

Next, we analyzed if this underrepresentation is homogeneous within the CDS. We divided the proteins into regions that are predicted to have an intrinsically ordered or disordered 3D structure [25]. As shown in Figure 1B, tandem acceptors are substantially underrepresented in ordered regions. Tandem donors exhibit the same trend but the underrepresentation in ordered regions is moderate (Figure 1A). This moderate underrepresentation might be due to differences in the frequency of polar and hydrophobic protein indels. The toleration of tandem donors in ordered regions might be higher, since they often insert hydrophobic residues (in particular Val [19]), which are preferred in ordered regions [25]. We also analyzed how frequently tandem sites affect other characteristic protein features such as Pfam domains, transmembrane (TM) helices, signal peptides, low complexity regions, coiled coil regions, and nuclear localization signals (NLS), which is described in detail in the Additional File 1 (see also Additional File 2). We found that low complexity regions are rather tolerant for tandem splice events (Additional File 3), which is consistent with observations for amino acid indels [26]. We also found evidence that tandem sites affecting TM helices, signal peptides, and NLS are selected against the insertion of particular amino acids (Additional File 1, 4, 5). Tandem sites are also significantly associated with specific DNA and RNA binding Pfam domains (Additional File 1, 6), consistent with reports for NAGNAG acceptors [4,27].

### Selection against a strong minor splice site in ordered regions

Using EST counts as a rough measure for the frequency of splicing at the minor splice site, we considered a minor splice site as strong if more than 25% of the ESTs support the minor splice variant and otherwise as weak (note that by definition >50% of the ESTs support the major variant). For tandem donors, only 33% of the 52 events with a strong minor site affect ordered regions compared to 57% of the 317 events with a weak site (Fisher's exact test: P = 0.0009). Likewise, for tandem acceptors, only 22% of the 454 events with a strong minor site affect ordered regions compared to 36% of the 1,312 events with a weak site (Fisher's exact test: P < 0.0001). Thus, ordered regions are under a strong selection against events that involve an efficient minor site.

### Location of tandem splice sites with respect to protein secondary structures

**Figure 2:**
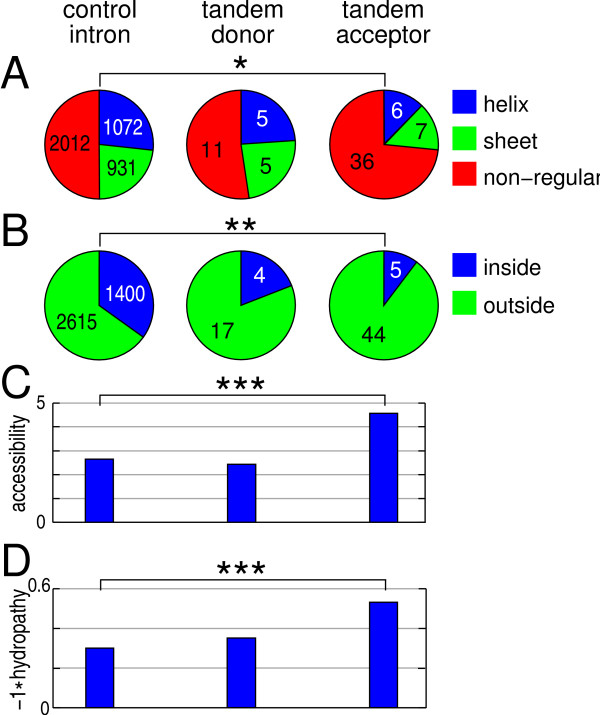
**Avoidance of tandem acceptors in structured regions of Pfam domains**. The distribution of exon/exon junctions derived from control introns, introns with tandem donors and acceptors (A) in alpha-helices, beta-sheets, and non-regular elements, (B) 'inside' or 'outside' structural elements (see text), (C) with respect to the average surface accessibility, and (D) with respect to the average inverse hydropathy scores. Kyte-Doolittle values were used to compute hydropathy scores for the ± 5 amino acid contexts. The values were inverted so that positive values indicate polar residues. To avoid potential biases, we excluded the insertion sequence of tandem donors and acceptors from the context. Different context lengths of ± 3, ± 10, or ± 15 residues give consistent results in D (Additional File 11). P-values using a χ^2 ^test in A and B and a Wilcoxon rank sum test in C and D are indicated as *: P < 0.05, **: P < 0.001, ***: P < 0.0001.

To further test the underrepresentation of tandem splice sites in ordered regions, we focused on Pfam domains since these domains usually fold into a well-defined 3D structure. We obtained the protein secondary structure as well as the surface accessibility of residues from known 3D structures of Pfam domains. We mapped the position of 21 and 49 introns with tandem donors and acceptors, respectively, as previously described [28]. For comparison, we mapped the position of 4,015 introns without a Δ3/Δ6/Δ9 tandem donor or acceptor motif (called control introns) since small in-frame splice site variations cannot occur in these introns. Comparing the location of introns with respect to alpha-helices, beta-sheets, and non-regular elements, we found no difference between control introns and introns with tandem donors. However, introns with tandem acceptors are significantly biased against a location in helices and sheets (Figure 2A). This tendency is even more pronounced for NAGNAG acceptors (Additional File 7). As the exact boundaries of structural elements are sometimes difficult to determine, we further analyzed a broader context of ± 1 residue around the intron location. We considered an intron to be 'inside a structural element' if this broader context is completely inside a helix or inside a sheet. If the complete context is inside a non-regular element or in two different structural elements, the context is considered to be 'outside a structural element'. In this comparison, both tandem donors and acceptors show a noticeable avoidance of structural elements (Figure 2B). The average surface accessibility scores are indistinguishable between control intron and tandem donor regions, while regions with tandem acceptors have a significantly higher surface accessibility (Figure 2C). Finally, we found polar residues to be slightly enriched in tandem donor and strongly enriched in tandem acceptor protein contexts (Figure 2D), which is further evidence that protein variations caused by tandem acceptors are preferentially located on the surface of folded domains.

### Strong bias against plausible NAGNAG acceptors in human CDS

To further investigate selection against tandem splice sites, we focused on NAGNAG acceptors for two reasons. First, these acceptors comprise the largest tandem class with a total of 7,835 acceptors. Secondly, the NAGNAG motif is highly predictive for alternative splicing [7,19,29]. That is, sites with the motif HAGHAG (H = A, C, or T, in the following called 'plausible' NAGNAG acceptors) are preferentially alternatively spliced (1,253 of 2,787; 45%). In contrast, only a small fraction of the acceptors with a HAGGAG, GAGHAG, or GAGGAG (called 'implausible') motif allow alternative splicing (143 of 5,048; 2.8%). Thus, we can use the motif to classify all NAGNAG acceptors into those that are likely and unlikely alternatively spliced, independent of available transcript data.

**Figure 3:**
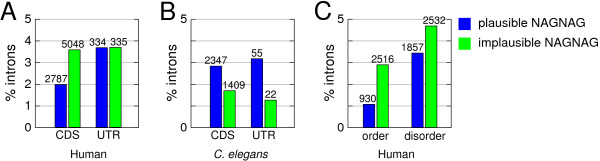
**Distribution of plausible and implausible NAGNAG acceptors**. (A) Human and (B) *C. elegans *UTR vs. CDS introns; (C) Human CDS introns divided into a location in ordered or disordered regions. Each bar is the percentage of introns having a plausible (blue) or implausible (green) NAGNAG acceptor. Absolute intron numbers are given above the bars.

First, we compared the percentage of CDS and UTR introns that have a plausible or implausible NAGNAG acceptor. The frequency of plausible NAGNAG sites is 1.9-fold lower in CDS introns compared to UTR introns (Figure 3A). In contrast, the frequency of implausible sites is very similar in CDS and UTR introns. This shows a significant depletion of plausible sites in CDS introns (Fisher's exact test: P < 0.0001). Consistently, AAG and CAG but not the synonymous codons AAA and CAA have been found to be avoided at the 5' exon boundary [30,31], although AAG/CAG is more often part of splicing enhancer motifs than AAA/CAA [32]. Furthermore, GAG is not underrepresented at the 5' exon boundary compared to the synonymous GAA codon [31].

To further test this, we considered NAGNAG sites in *C. elegans*, which is the only metazoan species found so far that lacks widespread alternative splicing at NAGNAG sites [4,19]. Two points indicate that the simple classification plausible/implausible is also valid for *C. elegans*. First, *C. elegans *acceptor AGs have exactly the same preference for the preceding nucleotide (C > T > A > G) as seen in mammals [33]. Secondly, all of the 33 alternatively spliced NAGNAG acceptors in *C. elegans *are plausible [19]. Thus, although the frequency of alternative splicing at NAGNAG sites is exceptionally low in *C. elegans *(20-fold lower compared to humans), those that are alternatively spliced have the same characteristics as in humans. Thus, we can use *C. elegans *as another control for the underrepresentation of plausible NAGNAG acceptors in human CDS introns. As expected, in *C. elegans*, we found that the frequency of plausible and implausible NAGNAG sites in UTR and CDS introns is very similar (Figure 3B). Interestingly, plausible NAGNAG acceptors are even more abundant than implausible ones both in UTR and CDS introns of *C. elegans*. This is presumably caused by a lower frequency for G at the 5' exon boundary in *C. elegans *(~25%) as opposed to humans (~50%) [33].

Next, we analyzed the frequency of NAGNAG sites in ordered and disordered regions. The frequency of introns with plausible NAGNAG sites is 3.2-fold lower in ordered compared to disordered regions. In contrast, the frequency of introns with implausible NAGNAG acceptors is only 1.6-fold lower in ordered regions (Figure 3C), which is a significant difference (Fisher's exact test: P < 0.0001). The frequency of NAGNAG sites in the other protein features is shown in Additional File 8.

**Figure 4:**
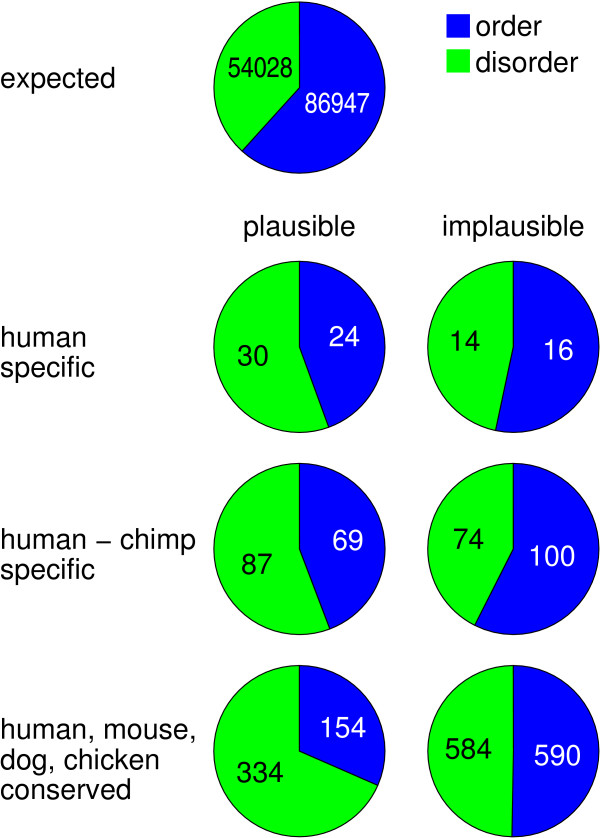
**Distribution of evolutionary 'young' and 'old' plausible vs. implausible NAGNAG acceptors in ordered and disordered regions**. The expected distribution is the overall number of introns in ordered/disordered regions.

Moreover, we analyzed the distribution of evolutionary 'young' and 'old' plausible/implausible NAGNAG acceptors in ordered/disordered regions. As shown in Figure 4, plausible NAGNAG sites that are human specific (not conserved in the orthologous chimpanzee, rhesus, and mouse introns) or human-chimpanzee specific (not conserved in rhesus and mouse) have a significant tendency to avoid ordered regions compared to the respective implausible ones (Fisher's exact test: P = 0.02, P < 0.0001, respectively). A stronger underrepresentation was observed for evolutionary old (conserved between human, mouse, dog, and chicken) plausible NAGNAG acceptors (Fisher's exact test: P < 0.0001). It is noteworthy that implausible NAGNAG acceptors resemble the expected distribution, which is the overall number of introns in ordered/disordered regions (Figure 4). Thus, young and old plausible NAGNAG sites are underrepresented in ordered regions, indicating that selection against such sites is universal and more effective over large evolutionary distances.

### Selection against NAGNAG creating mutations

**Figure 5:**
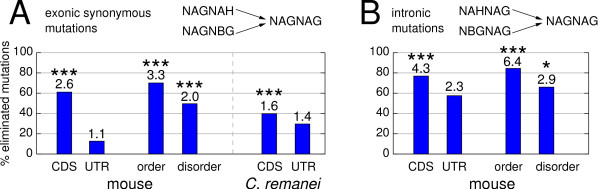
**The percentage of plausible NAGNAG creating mutations eliminated by selection**. (A) exonic and synonymous mutations, (B) intronic mutations. The relative risk (RR) indicating how much more likely the creation of an implausible NAGNAG is compared to the creation of a plausible NAGNAG is given above the columns. P-values of the CMH test are indicated as ***: P < 0.0001, **: P < 0.001, *: P < 0.01.

We have previously shown that a single nucleotide mutation can create an alternatively spliced NAGNAG acceptor [29]. We asked whether selection acts against single nucleotide substitutions creating plausible NAGNAG acceptors by comparing CDS with UTR as well as ordered with disordered regions. We considered only base exchanges that create a second AG dinucleotide in the context of a non-NAGNAG acceptor. To dissect selection against NAGNAG creations from other evolutionary pressures, we focused only on cases where the required mutation has to occur (i) within the exon and is synonymous (Figure 5A) or (ii) within the intron (Figure 5B). Controlling for different distributions in the required mutations and using implausible NAGNAG creations as a control (see Methods), we estimated the relative risk (RR) for the creation of an implausible vs. plausible NAGNAG acceptors using the Cochran-Mantel-Haenszel (CMH) test. An RR of 1 indicates that the creation of plausible and implausible NAGNAG sites is equally likely, which is consistent with the absence of selection, while an RR > 1 indicates how much more likely the creation of an implausible site is. Using the RR, we can estimate how many mutations that create plausible NAGNAG sites have been eliminated by selection.

First, we investigated the exonic creation of mouse NAGNAG sites inferred by comparing human acceptors with mouse and the outgroup dog. We found that the creation of an implausible NAGNAG site in the CDS is 2.6-fold more likely than the creation of a plausible NAGNAG, indicating that 62% of plausible NAGNAG creating mutations have been eliminated by selection (Figure 5A). In contrast, the creation of plausible/implausible acceptor motifs is equally likely in UTRs (RR = 1.1). In ordered regions, the RR of 3.3 (70% elimination) is substantially higher than the RR of 2 (50% elimination) in disordered regions (Figure 5A). This provides evidence for a selection pressure against the creation of plausible NAGNAG sites in the CDS, in particular against creations in ordered regions.

Next, we investigated NAGNAG creation by intronic mutations. The RR is higher for the CDS region compared to UTRs (Figure 5B). UTRs also exhibit an RR > 1, however the RR is estimated from sparse data and P = 0.11 does not suggest a significant deviation from the expected RR of 1. Consistent with our above results, ordered regions exhibit a noticeable higher RR than disordered regions (84 vs. 66% elimination). In general, intronic mutations that create plausible NAGNAG sites seem to be under stronger selection than exonic synonymous ones (compare Figure 5A with 5B). This might be explained by the fact that the upstream acceptor in a NAGNAG motif is generally the preferred splice site [3,4]. Thus, the creation of a new AG upstream of an existing acceptor is expected to result in the usage of the novel acceptor in the majority of transcripts. In contrast, a novel acceptor downstream should be the minor splice site, consequently the established transcript is predominantly expressed.

Since plausible NAGNAG sites in nematodes are rarely alternatively spliced [4,19], we would expect a much weaker or no selection against the creation of plausible NAGNAG sites. To test this, we examined the creation of NAGNAG sites in *C. remanei *using the genomes of *C. briggsae *and the outgroup *C. elegans*. Considering exonic synonymous mutations, CDS introns have an RR very similar to UTR introns (Figure 5A). While the RR for nematode CDS introns is significantly different from 1 (P < 0.0001), it is substantially lower than the RR for human CDS introns. This is consistent with a much weaker selection against plausible NAGNAG creations in nematode CDS. Nearly identical results were observed for NAGNAG creation in *C. briggsae *(Additional File 1). Compared to mammals, *C. elegans *introns have an unusual acceptor site that lacks a branch point consensus but contains a highly conserved TTTTCAG acceptor motif, which is a high affinity binding site for the U2AF heterodimer [34]. Deviations from this consensus were shown to affect U2AF binding [34]. Consistent with this, we found not a single case among 5,375 nematode introns where a NAGNAG is created by an intronic mutation.

## Discussion

We presented multiple lines of evidence that human coding sequences are under selection against in-frame tandem splice events. In particular, ordered regions are under strong selection, suggesting that even small changes (one to three amino acid differences) might affect protein folding and function. For example, the deletion of a few residues from the Piccolo C_2_A domain leads to a structural change with marked consequences for Ca^2+ ^binding [35]. We also found that tandem acceptor caused protein variations in functional domains are preferentially located at the surface and outside structural elements such as alpha-helices and beta-sheets. Similar results were reported for protein indels [26,36] and exon skipping events [28]. The frequent class of NAGNAG acceptors, which mostly result in the indel of only one amino acid [4], is also under strong evolutionary constraints. Consistently, a splicing-independent case study found phenotypic effects for two thirds of random triplet deletions [37].

Our findings of selection against tandem splice sites suggest that existing tandem sites are either (effectively) neutral or might have a functional role. Alternative splicing frequently affects protein regions that are intrinsically disordered [38]. Consistently, we found that tandem splice sites preferentially affect disordered regions. While alterations of disordered regions should not cause changes in protein structure in general, it can change the function of proteins [38], as disordered regions are often associated with regulation and signalling [39].

UTRs contain secondary structures and binding sites for proteins and non-coding RNAs that influence mRNA export, localization, stability as well as translational efficiency [40]. Thus, functional sites in UTRs are also expected to be under selection. However, our results on tandem sites in UTRs are consistent with neutral evolution, although individual introns can still be under selection against tandem sites.

## Conclusion

We found that CDS introns but not UTR introns are under selection against tandem sites. Thus, we can use the frequency of tandem sites in UTRs to provide a rough estimation of the number of CDS introns under selection against tandem splice sites. We found that 3.68% of the UTR and 1.98% of the CDS introns have a plausible NAGNAG (Figure 3). Under neutrality, we would expect that the percentage for CDS introns equals that for UTR introns, consequently we estimate 2,397 (1.7% = 3.68%–1.98%) of the 140,975 CDS introns to be under selection against a plausible NAGNAG acceptor. Likewise, we estimate that 536 (0.38% = 0.64%–0.26%, see Figure 1) and 1,819 (1.29% = 2.54%–1.25%) CDS introns are under selection against tandem donor and acceptor sites, respectively. In summary, we conclude that ~2,400 human introns are under selection against possessing a subtle tandem splice site inserting/deleting 3, 6, or 9 nt.

## Methods

### Data sets

We downloaded from the UCSC Genome Browser [41] the human genome assembly (hg17, May 2004) and the RefSeq annotation (refFlat.txt.gz, November 2006). We excluded all transcripts that have only one exon, that are candidates for NMD (stop codon >50 nt upstream of the last exon-exon junction), that lack a start or stop codon, or that have in-frame premature stop codons according to the RefSeq to genome mapping. Furthermore, transcripts with ambiguous characters were excluded. We also discarded transcripts that have exons shorter than 5 nt or introns shorter than 30 nt as they might have incorrect exon-intron structures. To get a set of non-redundant transcripts, we extracted RefSeq transcripts so that they do not overlap any other transcript on the same strand. To remove redundancy and strong similarity of the proteins encoded by a RefSeq transcript, we used NCBI BLASTClust to cluster the proteins by sequence similarity using 80% coverage and 80% identity (parameters -L 0.8 -S 80). Then, we kept only one protein from each cluster with more than one entry as well as all proteins from single clusters. This yields 15,511 non-redundant transcripts/proteins. For *C. elegans*, we downloaded the genome assembly ce2 (March 2004) and the RefSeq annotation (refFlat.txt.gz, May 2007). All transcripts were filtered as for human, except for omitting the NMD filter. We got 15,652 non-redundant *C. elegans *transcripts/proteins.

For all transcripts, we screened all splice sites for the presence of a tandem donor and acceptor Δ3, Δ6, and Δ9 motif. Annotated donors without GT/GC and acceptors without AG intron termini were omitted. The RefSeq annotation of the open reading frame was used to decide if a tandem site affects the CDS. A tandem site was considered as alternatively spliced if there is at least one EST/mRNA each that match the short and the long transcript. For Δ3 donor and acceptor sites, we downloaded EST information from TassDB [42]. For Δ6 and Δ9 tandem sites, we used BLAST against all ESTs and mRNAs. From the analyses, we omitted 35 introns where a Δ3/Δ6/Δ9 tandem splice event leads to the direct insertion of a stop codon (e.g. a CAGTAG acceptor in intron phase 0), since most of these events result in an NMD target and our aim is to analyze only splice events causing subtle mRNA and protein changes. Conservation of tandem sites was detected by analyzing the genome-wide pairwise alignments downloaded from the UCSC genome browser (assemblies: human hg17, chimpanzee panTro2, rhesus rheMac2, mouse mm7, dog canFam2, chicken galGal2) using the genomic locus of the human tandem sites to select the respective alignment chain.

We determined characteristic protein features for the protein sequence that corresponds to the annotated exon-intron structure. Given that the major splice site (inferred from EST counts) is annotated for 91.2% and 89.2% of the tandem donors and acceptors, respectively, the annotated exon-intron structure reflects the predominant protein isoform in the great majority of cases. Ordered and disordered regions were predicted by VSL2B [25]. See Additional File 9 for the other protein features.

### Location of tandem splice sites in Pfam domains structures

We considered all Pfam domains to which at least five tandem splice sites were mapped and that have a known 3D structure. As previously described [28], pdb2pfam was used to obtain the protein secondary structure assignment and the surface accessibility for each residue of a domain with known 3D structure (target domain). Then, we compared the Pfam alignment (the Viterbi path) of the target domain with the alignment of the query domain to map the position of the query intron. Specifically, we considered the exon-exon junction that represents this intron. If the exon-exon junction splits a codon, only this amino acid was marked as the exon junction. If the exon junction is located between two codons, we marked both neighboring amino acids. From the mapped exon-exon junction, we inferred the location in the secondary structure and the surface accessibility. We discarded cases where the exon-exon junction maps to an insert or delete state. For introns with tandem splice sites, we used the annotated exon-exon junction, since the annotated splice site is mostly the major site. The secondary structure assignment from the eight DSSP states was done as follows: H, G, I helix, E sheet, and T, S, C, B non-regular. The two states BC are converted to EE.

### NAGNAG creating mutations

We define as a 'pre-NAGNAG' acceptor a non-NAGNAG acceptor motif that requires a single base exchange to become a NAGNAG acceptor (e.g. AAACAG requires an A to G target mutation at position 3). Based on the nucleotide at the two N-positions, we distinguish between plausible and implausible pre-NAGNAG sites. For all human pre-NAGNAG acceptors, we determined the orthologous mouse and dog site using the genome-wide pairwise alignments from the UCSC genome browser [41]. Then, we considered the creation of a NAGNAG site in mouse, inferred by demanding that the outgroup dog has no NAGNAG acceptor. Cases of NAGNAG loss in human (i.e. NAGNAG in mouse and dog) were discarded. Based on the high sequence similarity between human and mouse proteins and highly similar exon-intron structures, a mouse intron should be located in the same region (CDS/UTR, order/disorder) as the human intron. We use the creation of implausible NAGNAG sites as a control, considering only mutations converting a non-AG into an AG dinucleotide (positions 2,3,5,6 in the pre-NAGNAG motif). Thus, the nucleotides at both N-positions do not affect our results, but they determine the likelihood for alternative splicing at the novel NAGNAG site. It is noteworthy that the comparison to implausible pre-NAGNAG sites controls for the general selection to preserve the polypyrimidine tract in case of intronic mutations, thus observed differences can be attributed to selection against plausible NAGNAG sites. As the six possible target mutations (C/G/T to A and A/C/T to G) are unequally distributed between plausible and implausible pre-NAGNAG sites (Additional File 10) and mutation rates differ (transitions are more frequent than transversions), we have to exclude these potential biases. Therefore, we estimated the relative risk (RR) for the gain of implausible vs. plausible NAGNAG acceptors using the Cochran-Mantel-Haenszel (CMH) test that corrects for the influence of the target mutation. The CMH test was computed using the SAS software. The RR is the ratio of the probability that an implausible NAGNAG is created and the probability that a plausible NAGNAG is created. To estimate what percentage of mutations that create plausible NAGNAG sites have been eliminated by selection, we computed (1-(1/RR))*100.

*C. elegans *is the only nematode with a sufficiently large transcript coverage and lacks widespread alternative splicing at NAGNAG acceptors. We assume that other nematodes also lack widespread alternative splicing at NAGNAG sites. This is supported by previous experimental observations that splicing regulation between both *C. elegans *and *C. briggsae *is highly conserved [43] and by a high conservation between *C. elegans *and *C. briggsae *acceptor sites [44]. Using the same procedure as above, we analyzed the *C. elegans *pre-NAGNAG acceptors using UCSC pairwise alignments of *C. elegans *(assembly ce4) with *C. briggsae *(cb3) and *C. remanei *(caeRem2) to infer NAGNAG creation in *C. briggsae *as well as *C. remanei *(note that *C. elegans *is the outgroup here [45]).

## Abbreviations

nt, nucleotides; NMD, nonsense-mediated mRNA decay; CDS, coding sequence; UTR, untranslated region; EST, expressed sequence tag; TM helix, transmembrane helix; NLS, nuclear localization signal; RR, relative risk; CMH test, Cochran-Mantel-Haenszel test.

## Authors' contributions

MH designed and performed all analyses, analyzed the data and drafted the manuscript. KS and KH contributed analysis tools. RB and MP were the principal investigators. All authors contributed to the final manuscript, read and approved it.

## Supplementary Material

Additional file 1 Supplementary Text. Click here for file

Additional file 2 Overlap between the protein features. Click here for file

Additional file 3 Frequency of tandem splice sites in different protein features. Click here for file

Additional file 4 Selection against insertion of particular amino acids. Click here for file

Additional file 5 Distribution of the insertion sequences of tandem sites in the protein features. Click here for file

Additional file 6 Association of tandem sites with specific Pfam domains or clans. Click here for file

Additional file 7 Avoidance of NAGNAG sites in structured regions of Pfam domains. Click here for file

Additional file 8 Distribution of plausible and implausible NAGNAG acceptors in different protein features. Click here for file

Additional file 9 Supplementary Methods Click here for file

Additional file 10 Unequal distribution of the target mutations between plausible and implausible human pre-NAGNAG sites and between CDS/UTR and order/disorder. Click here for file

Additional file 11 Average hydropathy scores for introns without tandem donor or acceptor motifs (control introns) and for introns with tandem donors and acceptors. Click here for file
