# Supplementary material for: Selection against tandem splice sites affecting structured protein regions
Source: BMC Evol Biol. 2008 Mar 21;8:89. doi: 10.1186/1471-2148-8-89 (PMC2279118; doi:10.1186/1471-2148-8-89)
Supplement: Additional file 1 — Supplementary Text. [file 1471-2148-8-89-S1.pdf]

Additional File 1: Supplementary Text

Inhomogeneous distribution of tandem sites in different protein regions

In addition to ordered and disordered regions (see main text), we annotated further characteristic features for the 15,511 proteins: functional protein domains (Pfam domains), transmembrane (TM) helices, signal peptides, low complexity regions, coiled coil regions, and nuclear localization signals (NLS) (Additional File 9). It should be noted that apart from ordered/disordered regions these protein features are neither disjunct nor cover the complete protein. That means an intron position can be assigned to either an ordered or disordered region, but for example can be assigned to an ordered region, a Pfam domain, and a TM helix. In general, Pfam domains, TM helices, and signal peptides have strong overlap with ordered regions, whereas NLS, coiled coils, and low complexity regions preferentially overlap with disordered regions (Additional File 2).

As shown in Additional File 3, the distribution of tandem donors and acceptors is not homogeneous in these protein features. The high frequency in low complexity regions suggest that these regions are rather tolerant for the effect of tandem splice events, consistent with a previous report of protein indels [1].

Context dependent selection against particular protein variations

TM helices and signal peptides are rich in hydrophobic amino acids (aa). NLS consist of stretches of positively charged residues. As tandem sites allow a variety but nevertheless limited set of different aa indels, we investigated the properties of the aa that occur only in the long protein variant (that arises by using the exon-distal splice site) with respect to the protein features. In TM domains and signal peptides, insertions of polar aa are significantly depleted ($P<0.05$ and $P<0.01$, respectively, Fisher´s exact test; Additional File 4A), while the frequency of polar aa in other protein features is close to the overall distribution (Additional File 5A). Consistently, in TM domains and signal peptides the average Kyte-Doolittle hydropathy score of the inserted residues is positive (indicating a hydrophobic nature), whereas tandem sites in general tend to insert polar aa (Additional File 4B). Furthermore, in TM helices and signal peptides, the average frequency of the splice variant that inserts a polar aa is slightly lower than the global average (69% and 66% vs. 73%, respectively; inferred from EST counts). For example, among all 14 cases of a CAGCAG acceptor that leads to the insertion of an Arg (the most polar aa), the single event that affects a TM helix has the lowest frequency observed (6%).

In NLS, positively charged aa are enriched, while negatively charged aa are significantly depleted ($P<0.05$, χ^2 test; Additional File 4C). This enrichment in positively charged aa is unique to NLS (Additional File 5B). Likewise, the average isoelectric point is higher compared to that of all events (Additional File 4D). The usage of the splice sites that insert the positively charged residues into NLS is higher than the global average (94% vs. 74%; inferred from EST counts). These results suggest that tandem sites affecting TM helices, signal peptides, and NLS are subject to selection against the

insertion of particular aa. However, we cannot exclude that these results are affected by an overall bias in specific aa in these regions.

Skewed distribution of tandem sites with specific Pfam domains

As described in Additional File 9, we analyzed the association of tandem donors and acceptors with specific Pfam domains or Pfam clans (functionally related Pfam domain families with a common evolutionary origin) [2]. We found that tandem sites are significantly depleted within the Collagen domain and the protein kinase clan (Additional File 6). On the other hand, tandem sites are significantly more frequent within the KRAB domain. We also found that tandem sites are significantly more frequently located up- or downstream of specific domains and clans, which have almost exclusively DNA or RNA binding activity. These associations are consistent with previous findings for NAGNAG sites [3, 4]. Overrepresentation might imply a function. Consistently, tandem splice events were observed to affect DNA binding and the ability to activate transcription [5, 6]. However, we cannot exclude that the overrepresentation is affected by constraints on the protein sequence in these domains or their adjacent regions, as the coding capacity at the exon boundaries and the propensity of certain tandem sites to be alternatively spliced are not independent.

NAGNAG creation by single nucleotide changes in *C. briggsae*

We assessed NAGNAG creation in *C. briggsae* by comparing acceptors of the outgroup *C. elegans* with *C. briggsae* and *C. remanei*. Concerning exonic synonymous mutations, CDS introns have an RR of 1.5 (P=0.0008), which is very similar to the observed value of 1.7 for *C. remanei* and substantially lower than the RR of 2.6 for human CDS introns. For UTR introns, we estimated an RR of 2 based on sparse data (not significant, P=0.31). Similarly to *C. remanei*, we found only a single case among 5,375 introns where a NAGNAG motif is created by an intronic mutation.

1. Taylor MS, Ponting CP, Copley RR: **Occurrence and consequences of coding sequence insertions and deletions in Mammalian genomes**. *Genome Res* 2004, **14**(4):555-566.
2. Finn RD, Mistry J, Schuster-Bockler B, Griffiths-Jones S, Hollich V, Lassmann T, Moxon S, Marshall M, Khanna A, Durbin R *et al*: **Pfam: clans, web tools and services**. *Nucleic Acids Res* 2006, **34**(Database issue):D247-251.
3. Akerman M, Mandel-Gutfreund Y: **Alternative splicing regulation at tandem 3' splice sites**. *Nucleic Acids Res* 2006, **34**(1):23-31.
4. Hiller M, Huse K, Szafranski K, Jahn N, Hampe J, Schreiber S, Backofen R, Platzer M: **Widespread occurrence of alternative splicing at NAGNAG acceptors contributes to proteome plasticity**. *Nat Genet* 2004, **36**(12):1255-1257.
5. Koenig Merediz SA, Schmidt M, Hoppe GJ, Alfken J, Meraro D, Levi BZ, Neubauer A, Wittig B: **Cloning of an interferon regulatory factor 2 isoform with different regulatory ability**. *Nucleic Acids Res* 2000, **28**(21):4219-4224.
6. Vogan KJ, Underhill DA, Gros P: **An alternative splicing event in the Pax-3 paired domain identifies the linker region as a key determinant of paired domain DNA-binding activity**. *Mol Cell Biol* 1996, **16**(12):6677-6686.
