# Supplementary material for: Selection against tandem splice sites affecting structured protein regions
Source: BMC Evol Biol. 2008 Mar 21;8:89. doi: 10.1186/1471-2148-8-89 (PMC2279118; doi:10.1186/1471-2148-8-89)
Supplement: Additional file 2 — Overlap between the protein features. [file 1471-2148-8-89-S2.pdf]

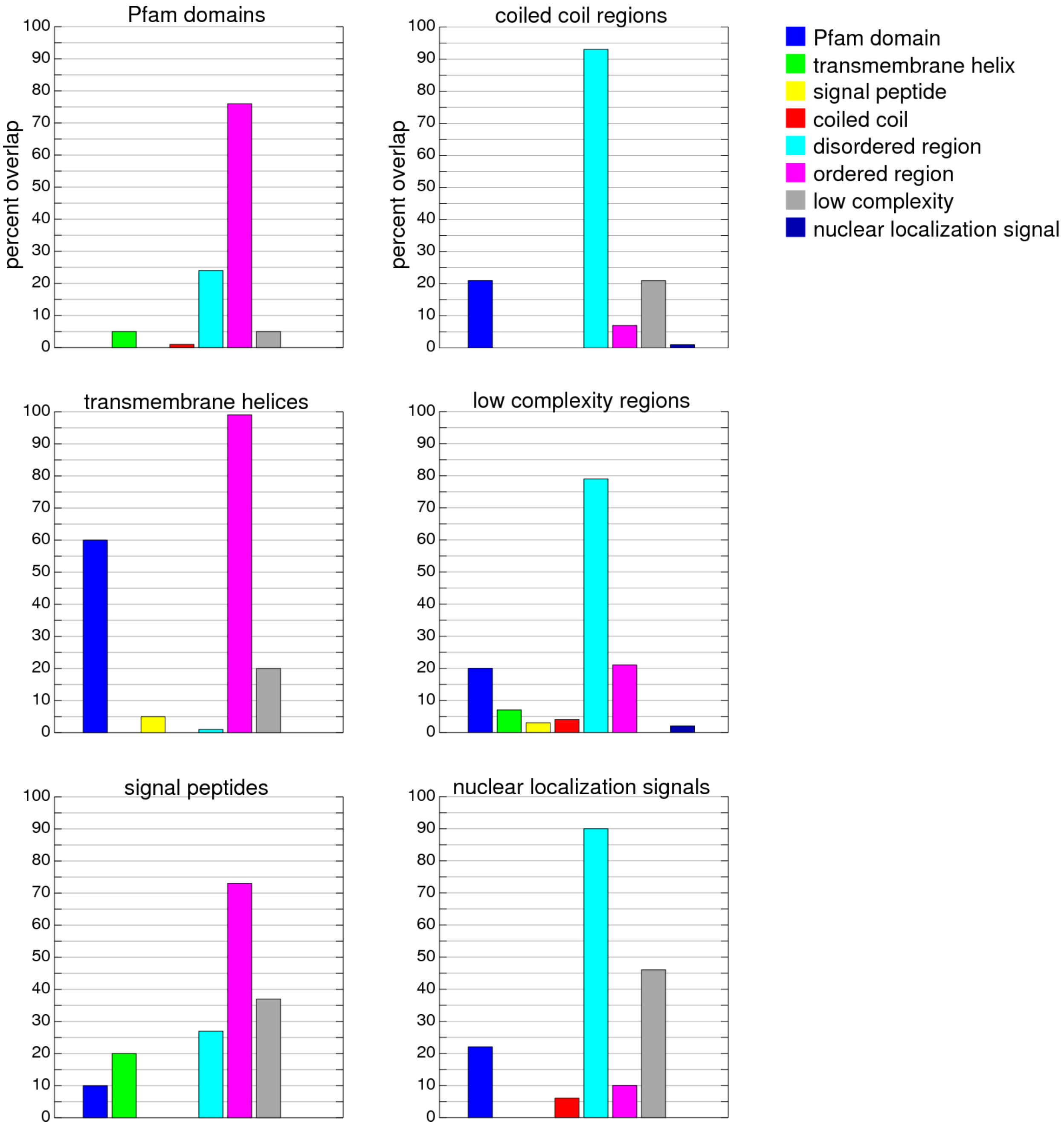

Additional File 2: Overlap between the protein features.

Each chart shows which percentage of residues in one protein feature overlap with another feature. Pfam domains, TM helices, and signal peptides have strong overlap with ordered regions, whereas coiled coil regions, low complexity regions, and NLS preferentially overlap disordered regions.
