# Supplementary material for: Selection against tandem splice sites affecting structured protein regions
Source: BMC Evol Biol. 2008 Mar 21;8:89. doi: 10.1186/1471-2148-8-89 (PMC2279118; doi:10.1186/1471-2148-8-89)
Supplement: Additional file 3 — Frequency of tandem splice sites in different protein features. [file 1471-2148-8-89-S3.pdf]

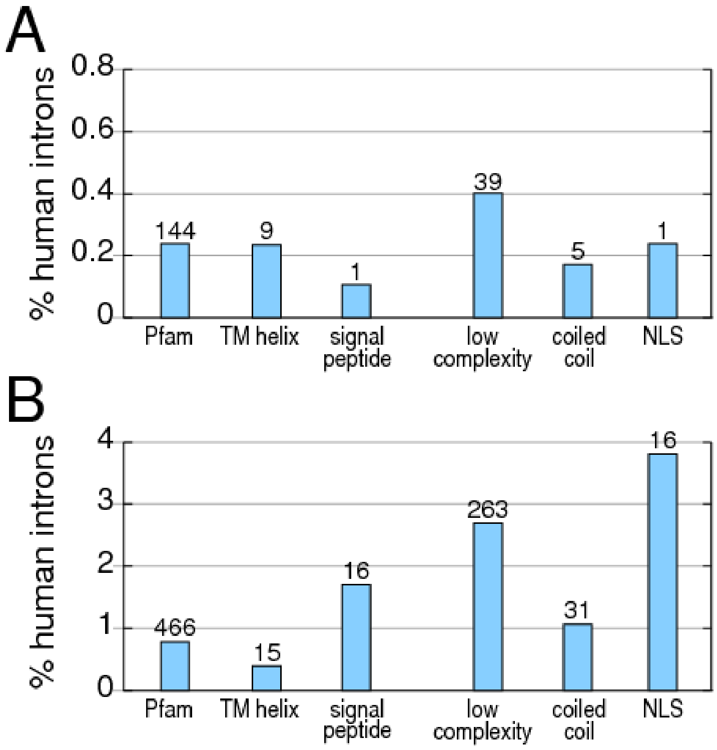

Additional File 3: Frequency of tandem splice sites in different protein features.

Each bar is the percentage of human introns having a tandem donor (A) or acceptor (B). CDS introns are divided into a location in the analyzed protein features. Absolute numbers are given above the bars.
