# Supplementary material for: Selection against tandem splice sites affecting structured protein regions
Source: BMC Evol Biol. 2008 Mar 21;8:89. doi: 10.1186/1471-2148-8-89 (PMC2279118; doi:10.1186/1471-2148-8-89)
Supplement: Additional file 4 — Selection against insertion of particular amino acids. [file 1471-2148-8-89-S4.pdf]

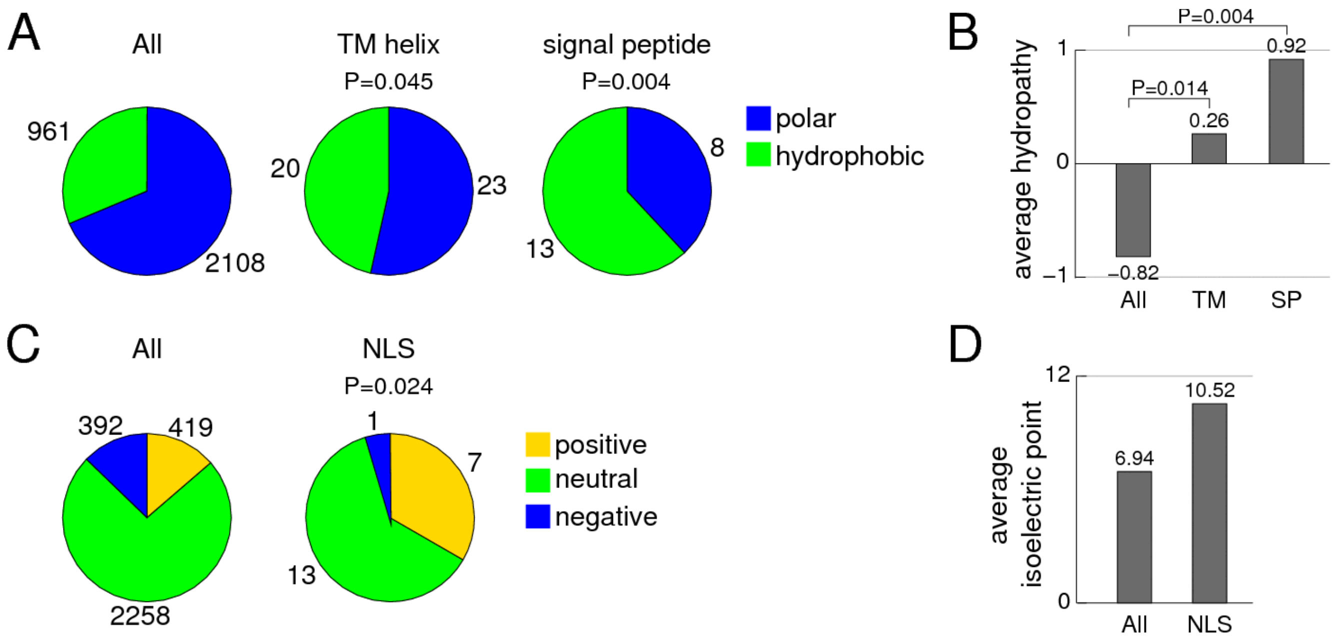

Additional File 4: Selection against insertion of particular amino acids.

(A) The distribution of polar and hydrophobic residues for all tandem sites and those that affect TM helices and signal peptides. (B) Average Kyte-Doolittle hydropathy score. Positive values stand for hydrophobic residues. SP = signal peptide. (C) Distribution of positively and negatively charged and neutral residues, plotted for all tandem sites and those that affect NLS. (D) Average isoelectric point. Absolute number of residues are given in A and C. P-values computed by Fisher´s exact test in A, Wilcoxon rank sum test in B, and χ^2 test in C are shown above the pie or bar charts. A Wilcoxon rank sum test in D gives a P-value of 0.0558, which is slightly higher than the significance threshold.
