# Supplementary material for: Selection against tandem splice sites affecting structured protein regions
Source: BMC Evol Biol. 2008 Mar 21;8:89. doi: 10.1186/1471-2148-8-89 (PMC2279118; doi:10.1186/1471-2148-8-89)
Supplement: Additional file 5 — Distribution of the insertion sequences of tandem sites in the protein features. [file 1471-2148-8-89-S5.pdf]

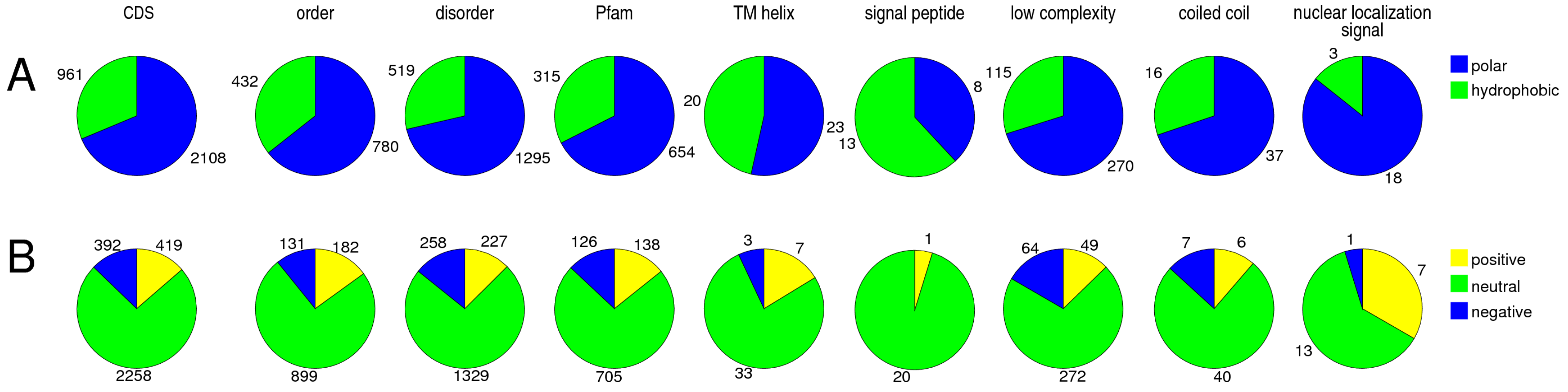

Additional File 5: Distribution of the insertion sequences of tandem sites in the protein features.

(A) The distribution of polar and hydrophobic residues and (B) positively and negatively charged and neutral residues, plotted for all tandem sites (CDS) and tandems that affect different protein features. Numbers give the absolute number of residues. The enrichment in polar residues in nuclear localization signals is not significant (P=0.1, Fisher´s exact test) and the different distribution in charged residues in signal peptides is also not significant (P=0.072, χ^2 test).
