# Supplementary material for: Selection against tandem splice sites affecting structured protein regions
Source: BMC Evol Biol. 2008 Mar 21;8:89. doi: 10.1186/1471-2148-8-89 (PMC2279118; doi:10.1186/1471-2148-8-89)
Supplement: Additional file 6 — Association of tandem sites with specific Pfam domains or clans. [file 1471-2148-8-89-S6.pdf]

Additional File 6: Association of tandem sites with specific Pfam domains or clans.

Pfam / Clan ID	description	total number [a]	expected [b]	observed [c]	P-value [d]
inside Pfam					
PF01391	Collagen triple helix repeat	1,148	17	0	<0.0001
PF01352	KRAB box	239	4	16	0.0019
inside CLAN					
CL0016	Protein kinase superfamily	2,518	38	14	0.0023
(up/down/between) Pfams					
PF00096	Zinc finger, C2H2 type	1,541	23	48	0.0069
PF01352	KRAB box	501	8	24	0.0016
PF00249	Myb-like DNA-binding domain	319	5	17	0.0141
PF00076	RNA recognition motif	1,140	17	63	<0.0001
PF00533	BRCA1 C Terminus (BRCT) domain	161	2	12	0.0103
PF00125	Core histone H2A/H2B/H3/H4	22	0	5	0.0235
(up/down/between) Clans					
CL0114	HMG-box like superfamily	222	3	12	0.028
CL0221	RRM-like clan	1,155	17	64	<0.0001

[a] total number of introns that are associated with the domain or clan

[b] number of introns * percentage of introns with tandem sites, which is 1.51% (2,135 tandems in 140,975 introns)

[c] observed number of introns with tandem sites

[d] Binomial test, after Bonferroni correction for multiple testing
