# Supplementary material for: Selection against tandem splice sites affecting structured protein regions
Source: BMC Evol Biol. 2008 Mar 21;8:89. doi: 10.1186/1471-2148-8-89 (PMC2279118; doi:10.1186/1471-2148-8-89)
Supplement: Additional file 7 — Avoidance of NAGNAG sites in structured regions of Pfam domains. [file 1471-2148-8-89-S7.pdf]

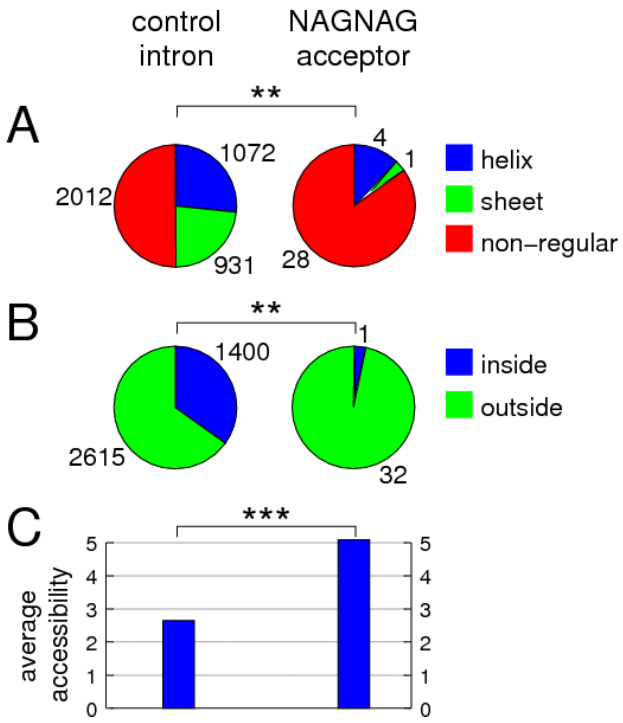

Additional File 7: Avoidance of NAGNAG sites in structured regions of Pfam domains.

The distribution of exon-exon junctions derived from control introns (introns without a Δ3/Δ6/Δ9 tandem donor or acceptor motif) and introns with NAGNAG acceptors (A) in alpha-helices, beta-sheets, and non-regular elements, (B) 'inside' or 'outside' structural elements (see main text), and (C) with respect to the surface accessibility. P-values using a χ^2 test in A and B and a Wilcoxon rank sum test in C are indicated as **: P<0.001, ***: P<0.0001.
