# Supplementary material for: Selection against tandem splice sites affecting structured protein regions
Source: BMC Evol Biol. 2008 Mar 21;8:89. doi: 10.1186/1471-2148-8-89 (PMC2279118; doi:10.1186/1471-2148-8-89)
Supplement: Additional file 8 — Distribution of plausible and implausible NAGNAG acceptors in different protein features. [file 1471-2148-8-89-S8.pdf]

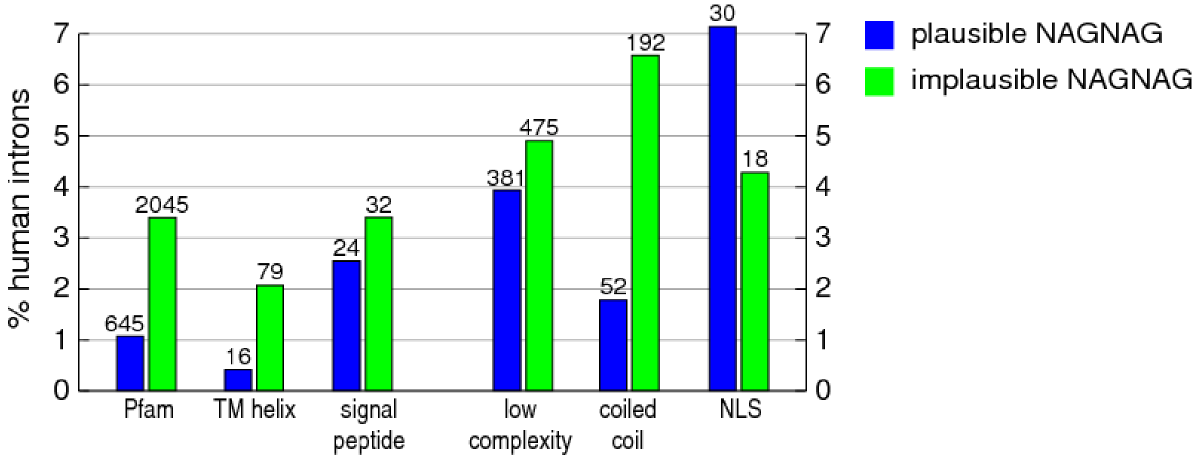

Additional File 8: Distribution of plausible and implausible NAGNAG acceptors in different protein features.

The figure shows that apart from disordered regions (Figure 3, main text), plausible NAGNAG sites are also frequent in signal peptides, low complexity regions, and NLS. The exceptional high frequency of plausible NAGNAG sites in NLS is caused by many HAGAAG sites in intron phase 0 introducing the positively charged Lys, which occurs frequently in NLS. The high frequency of implausible NAGNAG sites in coiled coils is caused by numerous NAGGAG sites in phase 0 encoding a Glu residue.
