# Supplementary material for: Selection against tandem splice sites affecting structured protein regions
Source: BMC Evol Biol. 2008 Mar 21;8:89. doi: 10.1186/1471-2148-8-89 (PMC2279118; doi:10.1186/1471-2148-8-89)
Supplement: Additional file 9 — Supplementary Methods [file 1471-2148-8-89-S9.pdf]

Additional File 9: Supplementary Methods

Annotation of protein features

Pfam domains were annotated using hmmpfam from the HMMER package (http://hmmer.wustl.edu/) with gathering cut-offs and the Pfam database version 20 (Pfam_fs.gz) [1]. Signal peptides were annotated using the SignalP 3.0 web server (http://www.cbs.dtu.dk/services/SignalP/) [2], transmembrane helices using the TMHMM web server (http://www.cbs.dtu.dk/services/TMHMM/) [3], low complexity regions using the programs SEG and XNU (parameter -p 0.001) included in the WU-BLAST package (http://blast.wustl.edu/), NLS using PredictNLS 1.3 [4], and coiled coil regions using MARCOIL 1.2 with a 99% threshold [5].

Association of tandem sites with specific Pfam domains

We determined all introns that are located within a specific Pfam domain or clan (functionally related Pfam domain families with a common evolutionary origin). An intron is located up- or downstream of a domain if it is in the linker region between two domains or between the C- or N-terminus and this domain. We used a Binomial test to assess if the frequency of tandem sites is significantly different in these introns compared to 1.51%, which is expected from the global frequency of tandem sites in the CDS.

1. Finn RD, Mistry J, Schuster-Bockler B, Griffiths-Jones S, Hollich V, Lassmann T, Moxon S, Marshall M, Khanna A, Durbin R *et al*: **Pfam: clans, web tools and services**. *Nucleic Acids Res* 2006, **34**(Database issue):D247-251.
2. Bendtsen JD, Nielsen H, von Heijne G, Brunak S: **Improved prediction of signal peptides: SignalP 3.0**. *J Mol Biol* 2004, **340**(4):783-795.
3. Krogh A, Larsson B, von Heijne G, Sonnhammer EL: **Predicting transmembrane protein topology with a hidden Markov model: application to complete genomes**. *J Mol Biol* 2001, **305**(3):567-580.
4. Cokol M, Nair R, Rost B: **Finding nuclear localization signals**. *EMBO Rep* 2000, **1**(5):411-415.
5. Delorenzi M, Speed T: **An HMM model for coiled-coil domains and a comparison with PSSM-based predictions**. *Bioinformatics* 2002, **18**(4):617-625.
