# Supplementary material for: Selection against tandem splice sites affecting structured protein regions
Source: BMC Evol Biol. 2008 Mar 21;8:89. doi: 10.1186/1471-2148-8-89 (PMC2279118; doi:10.1186/1471-2148-8-89)
Supplement: Additional file 10 — Unequal distribution of the target mutations between plausible and implausible human pre-NAGNAG sites and between CDS/UTR and order/disorder. [file 1471-2148-8-89-S10.pdf]

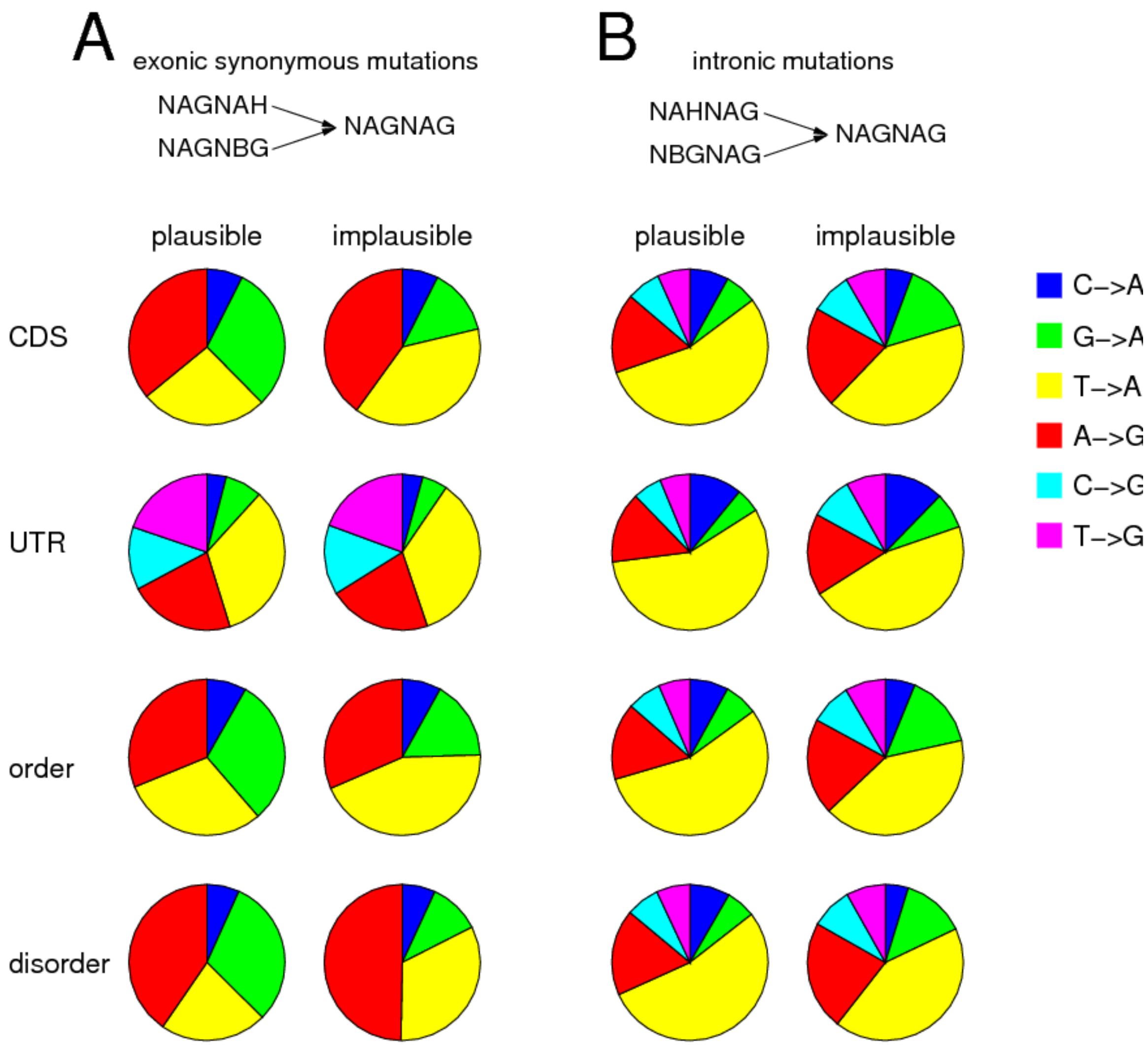

Additional File 10: Unequal distribution of the target mutations between plausible and implausible human pre-NAGNAG sites and between CDS/UTR and order/disorder.

(A) exonic synonymous mutations, (B) intronic mutations. Note that C->G and T->G target mutations are never synonymous in (A).
