# Supplementary material for: Selection against tandem splice sites affecting structured protein regions
Source: BMC Evol Biol. 2008 Mar 21;8:89. doi: 10.1186/1471-2148-8-89 (PMC2279118; doi:10.1186/1471-2148-8-89)
Supplement: Additional file 11 — Average hydropathy scores for introns without tandem donor or acceptor motifs (control introns) and for introns with tandem donors and acceptors. [file 1471-2148-8-89-S11.pdf]

Additional File 11: Average hydropathy scores for introns without tandem donor or acceptor motifs (control introns) and for introns with tandem donors and acceptors.

	control introns	tandem donor		tandem acceptor	
context length	average hydropathy	average hydropathy	P-value [a]	average hydropathy	P-value [a]
±3	-0.36	-0.43	0.58	-0.59	<0.0001
±5	-0.30	-0.35	0.47	-0.53	<0.0001
±10	-0.26	-0.32	0.14	-0.44	<0.0001
±15	-0.26	-0.25	0.97	-0.40	0.0016

[a] Wilcoxon rank sum test comparing control introns with tandem donors/acceptors

To avoid potential biases, we excluded the insertion sequence of tandem donors and acceptors from the context.
